# In situ approaches show the limitation of the spoilage potential of *Juniperus phoenicea* L. essential oil against cold-tolerant *Pseudomonas fluorescens* KM24

**DOI:** 10.1007/s00253-021-11338-3

**Published:** 2021-05-14

**Authors:** Kamila Myszka, Natalia Tomaś, Łukasz Wolko, Artur Szwengiel, Anna Grygier, Katarzyna Nuc, Małgorzata Majcher

**Affiliations:** 1 Department of Biotechnology and Food Microbiology, Poznan University of Life Sciences, Wojska Polskiego 48, PL-60,627 Poznan, Poland; 2 Department of Biochemistry and Biotechnology, Poznan University of Life Sciences, Dojazd 11, PL-60-632 Poznan, Poland; 3 Department of Food Technology of Plant Origin, Poznan University of Life Sciences, Wojska Polskiego 31, PL-60-624 Poznan, Poland

**Keywords:** *Juniperus phoenicea* L. essential oil, Quorum sensing, *Pseudomonas fluorescens*, Spoilage of seafood

## Abstract

**Abstract:**

The present study aimed to elucidate the effect of subinhibitory concentrations (sub-MICs) of juniper essential oil (EO), α-pinene, and sabinene on the quorum-sensing (QS)–mediated proteolytic and lipolytic properties of *Pseudomonas fluorescens* KM24. These activities were verified under in situ conditions, in which sub-MICs of the agents altered the morphology of KM24 cells. RNA-Seq studies revealed key coding sequences (CDSs)/genes related to QS and the proteolytic/lipolytic activities of pseudomonads. In this work, all the examined agents decreased autoinducer synthesis and influenced the mRNA expression of the encoding acyltransferase genes *lptA*, *lptD*, and *plsB*. The highest reduction on the 3^rd^ and 5^th^ days of cultivation was observed for the genes *lptD* (−5.5 and −5.61, respectively) and *lptA* (−3.5 and −4.0, respectively) following treatment with EO. Inhibition of the *lptA*, *lptD*, and *plsB* genes by singular constituents of EO was on average, from −0.4 to −0.7. At 5 days of cultivation the profile of AHLs of the reference *P. fluorescens* KM24 strain consisted of 3-oxo-C14-HSL, 3-oxo-C6-HSL, C4-HSL, and *N*-[(RS)-3-hydroxybutyryl]-HSL, the concentrations of which were 0.570, 0.018, 3.744, and 0.554 μg ml^−1^, respectively. Independent of the incubation time, EO, α-pinene, and sabinene also suppressed the protease genes *prlC* (−1.5, −0.5, and −0.5, respectively) and *ctpB* (−1.5, −0.7, and −0.4, respectively). Lipolysis and transcription of the *lipA*/*lipB* genes were downregulated by the agents on average from −0.3 to −0.6. α-Pinene- and sabinene-rich juniper EO acts as an anti-quorum-sensing agent and can repress the spoilage phenotype of pseudomonads.

**Key points:**

- *Juniper EO, α-pinene, sabinene exhibited anti-QS potential toward KM24.*
- *RNA-Seq revealed key CDSs/genes related to QS/proteolytic/lipolytic activities of KM24.*
- *Agents at sub-MIC levels influenced the mRNA expression of QS/lipase/protease genes.*

**Supplementary Information:**

The online version contains supplementary material available at 10.1007/s00253-021-11338-3.

## Introduction

Spoilage of seafood during refrigerated storage is primarily due to autolysis, bacterial growth, and metabolic activities, which result in the formation of off-flavor compounds, and chemical oxidation of lipids. However, the synthesis of proteases and lipases by contaminated microflora is by far the most important factor decreasing seafood quality (Ackman 1994; Xie et al. 2018). Extracellular secreted proteases are free to degrade nitrogenous substrates, thereby leading to extensive damage to food constituents and the production of ammonia, trimethylamine, hydrogen sulfide, hexanal, and 2,4-heptadienal (Haard 1994). Microbial lipases cause the production of off-flavor esters (Ackman 1994). All these factors lead to increased weight loss, reduced water-retention capacity, textural changes, and off-odor in fish-based products (Ackman 1994; Haard 1994). Approximately 10% of fish-based products are estimated to be lost each year as a result of microbial growth and proteolytic/lipolytic metabolism. Moreover, excessive spoilage of fish increases the overexploitation of natural resources to meet the global demand for fish-based products (Love et al. 2015).

Recent studies have shown that cold-tolerant *Pseudomonas* spp., mostly *Pseudomonas fluorescens*, can cause premature deterioration of seafood; these strains are dominant in sardine-, mackerel-, and salmon-based products under cold chain logistics (Venugopal 1990; Myszka et al. 2020). Spoilage activities of *P. fluorescens* within fish ecosystems may lead to the denaturation of muscle proteins, degradation of lipids, and structural damage to the fish membranes (Ackman 1994; Xie et al. 2018). Although pseudomonads are confirmed to decrease seafood quality, no systematic analysis of the spoilage activities of cold-tolerant *P. fluorescens* within food ecosystems has been performed to date. The mapping of molecular constituents in cells by high-throughput transcriptome sequencing technology (RNA-Seq) will contribute to our in-depth understanding of *P. fluorescens* spoilage activities in fish-based products and indicate key coding sequences (CDSs)/genes with altered expression that lead to better inactivation of bacteria within food. This information may help to develop an effective strategy to combat spoilage microflora and/or verify the actual effect of novel/alternative biocides on *P. fluorescens* cells.

Our previous work revealed that pseudomonads under refrigeration conditions can synthesize autoinducers of quorum-sensing (QS) systems (Myszka et al. 2020; Sobieszczańska et al. 2020). Autoinducers can interact with cognate receptor proteins, which leads to the expression of specific genes that regulate the spoilage activities of cells (Papenfort and Bassler 2016). Signal molecules of pseudomonads belong to the *N*-acyl-homoserine lactone (AHL) group; blockade of AHL synthesis can be regarded as a new preservation technique for fish-based products, potentially changing the expression level of genes related to the bacterial synthesis of lipases and proteases (Lee and Zhang 2015). Various chemicals for QS attenuation have been suggested, but only plant-derived essential oils (EOs) at subinhibitory concentrations (sub-MICs) meet the requirements of modern preservatives. Sub-MIC EOs can also be used as valuable flavoring and aromatizing seafood additives. The seafood to which EOs were added for flavoring purposes exhibits high microbial quality overall (Falleh et al. 2020). Recent reports have demonstrated that certain bioactive compounds from juniper EO are capable of repressing the synthesis of QS autoinducers; additionally, seasoning fish with juniper increases the sensory appeal of the products (Montagne 1999). However, it remains unclear how these agents influence the QS-modulatory properties of *P. fluorescens* within fish-based products.

This study aimed to elucidate the effect of sub-MICs of juniper EO and its major bioactive compounds on the QS-mediated proteolytic and lipolytic activities of *P. fluorescens* KM24. This work verified the following hypotheses: (i) sub-MICs of juniper EO and its major constituents changed QS autoinducer production and (ii) sub-MICs of juniper EO and its major constituents downregulated the expression levels of genes encoding lipases and proteases, and therefore decreased the proteolytic and lipolytic activities of pseudomonads. In this study, the global *P. fluorescens* transcriptome was characterized for the first time, which indicated the expression of all the evaluated genes was related to AHL synthesis and the proteolytic/lipolytic activities of KM24 in situ. Scanning electron microscopy (SEM) observations were performed to evaluate the KM24 morphology under the influence of the tested antimicrobials.

## Materials and methods

### Microorganisms

The KM24 strain was isolated from commercially available fresh salmon (*Salmo salar*) fillets. Sequencing and analysis of restriction length polymorphisms of the 16S rRNA gene amplicon were carried out for strain identification. The strain was deposited at the Polish Collection of Microorganisms (WDCM 106) (Wroclaw, Poland) under the number PCM3107.

*Chromobacterium violaceum* CV026 (from the National Collection of Type Cultures, Salisbury, UK) was used as a biosensor strain to measure the level of AHL production by KM24. CV026 is a violacein and AHL-negative double mini-Tn5 mutant of the ATCC 31532 strain.

Cryovials (Medical Wire and Equipment, Corsham, UK) were used for the preservation of strains.

### In situ cultivation

A fish juice medium obtained from fresh salmon fillets according to our previous work (Sobieszczańska et al. 2020) was developed for cultivation purposes. The medium was enriched with 0.10 M phosphate buffer, 0.065 M H_2_KPO_4_, and 0.044 M HK_2_PO_4_ (POCH, Gliwice, Poland). The pH value of the medium was adjusted to 6.6. After sterilization, the medium was supplemented with 1.6 g/L trimethylamine *N*-oxide, 40 mg/L L-cysteine, and 40 mg/L L-methionine (Sigma-Aldrich, Merck KGaA, Saint Louis, USA).

Total lipid extraction in the fish juice medium was performed according to the modified Cvrtila et al. (2004) method. Briefly, 50 ml of the medium was placed in a 300-ml Erlenmeyer flask; then, 15 ml HCl (Sigma-Aldrich, Merck KGaA, Saint Louis, USA) was accurately pipetted into the flask, and the flask was connected to a reflux condenser. The mixture was boiled for 15 min. Then, 10 ml anhydrous ethanol (POCH, Gliwice, Poland) and 50 ml petroleum ether (Sigma-Aldrich, Merck KGaA, Saint Louis, USA) were added. The content of the flask was shaken and allowed to settle, after which 15 ml was pipetted off, passed through a cotton-wool guard plug, and filtered into a tared receiver. The sample was boiled in a water bath and then dried at 105 °C. The sample was reweighed.

The Bradford assay was used for estimation of protein in the fresh juice medium (Bradford 1976). A standard curve was generated for bovine serum albumin (Sigma-Aldrich, Merck KGaA, Saint Louis, USA), and the absorbance at 595 nm was read.

Fish juice media supplemented with selected concentrations of juniper EO and its major bioactive compounds were inoculated with KM24. The cultivation processes were carried out at 4 °C ± 2 °C for 5 days.

### Whole-transcriptomic analysis (RNA-Seq) of KM24

Total RNA from KM24 cells after 5 days of cultivation was isolated using an RNAqueous Kit (Thermo Fisher Scientific, Waltham, MA, USA) following the manufacturer’s instructions. Excess ribosomal RNA was removed from the samples using a Ribominus Transcriptome Isolation Kit (Invitrogen, Carlsbad, CA, USA). Libraries were constructed using the Collibri^TM^ Stranded RNA Library Prep Kit from Illumina^TM^ and the Collibri^TM^ H/M/R rRNA Depletion Kit (Invitrogen, Carlsbad, CA, USA). Before sequencing, the libraries were quantitatively and qualitatively assessed using a Qubit fluorimeter (Thermo Fisher Scientific, Waltham, MA, USA) and a Bioanalyzer DNA electropherogram (Agilent, Santa Clara, CA, USA). Next-generation sequencing (NGS) was conducted with a MiSeq Illumina sequencer using the MiSeq Reagent Kit v3 (150 cycles) (Illumina, Hayward, CA, USA). The sequence reads were processed using CLC Genomic Workbench v20 (Qiagen, Valencia, CA, USA). The reads were mapped to the appropriate reference bacterial genome (NZ_CP049044) with gene and CDS and Pfam annotations. The results were normalized by counting the fragments per kilobase million (FPKM) values. RNA-Seq data were deposited in the SRA NCBI data repository (Bioproject: PRJNA509367; Biosample: SRX9799403; SRA: SRR1337604).

### Juniper essential oil extraction and identification of compounds

Juniper EO was extracted from dried berries of juniper (*Juniperus phoenicea* L.) from Italy by the hydrodistillation method in a Clevenger-type apparatus. Chemical characterization of juniper EO was further carried out by a Hewlett-Packard HP 7890A gas chromatograph coupled to a 5975C mass spectrometer (Agilent, Santa Clara, CA, USA) according to our previous study (Myszka et al. 2020). Mass spectra were obtained at 70 eV in the mass scanning range of 33–350 m/z. Compounds of juniper EO were identified by comparing the mass spectra and peak retention indices (RIs) with those of standards of homologous series of *n*-alkanes (C7–C24) under the same operating conditions. The relative percentages of the specific compounds were determined based on the gas chromatography peak areas relative to the total EO.

### Sub-MIC determination

The sub-MICs of juniper EO and major compounds against KM24 were evaluated using a broth macrodilution method according to the Clinical and Laboratory Standards Institute (2012). Juniper EO and its major compounds were diluted in dimethyl sulfoxide (DMSO) (Sigma-Aldrich, Merck KGaA, Saint Louis, USA) to obtain a range of concentrations. Uninoculated fish juice medium without juniper EO, α-pinene, and sabinene (Sigma-Aldrich, Merck KGaA, Saint Louis, USA) served as the controls. Microbial growth was assessed during incubation at 4 °C. The concentrations resulting in no significant growth inhibition were selected as sub-MICs.

### Morphology analysis of KM24

The morphology of KM24 after treatment with sub-MICs of juniper EO, α-pinene, and sabinene (Sigma-Aldrich, Merck KGaA, Saint Louis, USA) was evaluated by SEM (SU3500 Hitachi High-Technologies Corporation, Tokyo, Japan). After 5 days of cultivation, one drop of KM24 culture was deposited on a carbon sticker, air-dried, and coated with gold. For observations of samples, a low voltage (15 kV) was used.

### Extraction and quantification/qualification of AHLs

The method of Ravn et al. (2001) was used to extract QS autoinducers synthesized by KM24 in media supplemented with sub-MIC concentrations of juniper EO, α-pinene, and sabinene (Sigma-Aldrich, Merck KGaA, Saint Louis, USA). Briefly, cultures were centrifuged (3000 *g* for 10 min), and the supernatants were filtered through a Millex-GP 0.22-μm filter (Sigma-Aldrich, Merck KGaA, Saint Louis, USA). The supernatants were extracted with ethyl acetate (POCH, Gliwice, Poland), and the extracts were concentrated by rotary evaporation.

The method described by Blosser and Gray (2000) was used for rapid screening of the synthesis of AHLs by KM24. CV026 was grown overnight in TYB medium (BD, New Jersey, USA) and diluted in sterile medium to an optical density (540 nm) of 0.2. Extracts of AHLs were redissolved in 1 ml of the CV026 culture suspension. Samples were then incubated at 30 °C for 16 h. Next, the cells were lysed by adding 200 μl of 0.34 M sodium dodecyl sulfate (Sigma-Aldrich, Merck KGaA, Saint Louis, USA), vortexing, and incubating at room temperature for 5 min. Violacein was quantitatively extracted from the cell lysate by adding 900 μl of butanol (Sigma-Aldrich, Merck KGaA, Saint Louis, USA). The absorbance of the butanol phase was read at 585 nm on a SPECORD® 205 UV-VIS spectrophotometer (Analytic Jena AG, Jena, Germany). The CV026 cell response of the AHL extract was calculated according to formula (1):

1 $$ CVO26\  responce\ of\  AHL=\left(A/B\right)\times 1000 $$

where *A* is the absorbance of the butanol extract of violacein and *B* is the CV026 cell density.

The values obtained from the calculations are referred to as violacein units (UVs).

AHL molecules were also identified by a Dionex UltiMate 3000 (Thermo Fisher Scientific, Waltham, MA, USA) RP-UHPLC chromatograph coupled with ESI-MS with a qTOF system using a Kinetex^TM^ 1.7 Mm C18 100 Å LC column with dimensions of 100 × 2.1 mm (Phenomenex, Torrance, USA) according to our previous study (Myszka et al. 2020). Mass spectra were recorded in a scan range of *m/z* 80–1200. Data Analysis 4.1 software (Bruker Daltonik, Bremen, Germany) was used for LC-MS data processing. The molecular ions [M + H]^+^ and sodium adducts [M + Na]^+^ in positive ion mode were extracted from full-scan chromatograms. AHL molecules were identified by comparing the retention times of the samples with those of standards based on molecular mass information from the MS detector and MSMS data. Quantitative analyses of AHLs were conducted with calibration samples prepared in methanol as a surrogate matrix. The limits of detection (LODs) (S/N < 3) for the evaluated molecules were as follows: 0.04 μg ml^−1^ for C4-HSL and *N*-[(RS) – 3-hydroxybutyryl]-HSL and 0.005 μg ml^−1^ for 3-oxo-C14-HSL.

### Proteolytic and lipolytic activity estimation

The method proposed by Polychroniadou (1988) was used for estimation of KM24 proteolytic activity after treatment with sub-MICs of juniper EO, α-pinene, and sabinene (Sigma-Aldrich, Merck KGaA, Saint Louis, USA) with some modifications. Briefly, 0.5 ml of a pseudomonad culture was mixed with 0.5 ml of buffer consisting of 0.1 M Na_2_B_4_O_7_ in 0.1 M NaOH (POCH, Gliwice, Poland). Next, 1 ml (1 mg/ml) of 2,4,6-trinitrobenzenesulfonic acid (TNBS) (Sigma-Aldrich, Merck KGaA, Saint Louis, USA) was added. After incubation of the samples at 37 °C for 60 min, 2 ml of 0.1 M NaH_2_PO_4_ (POCH, Gliwice, Poland) containing 1.5 ml Na_2_SO_3_ (POCH, Gliwice, Poland) was added. The samples prepared with 0.5 ml of H_2_O served as controls. The absorbance at 420 nm on a SPECORD® 205 UV-VIS spectrophotometer (Analytic Jena AG, Jena, Germany) was read. The percentage of proteolytic activity inhibition was calculated according to the following formula (2):

2 $$ Proteolytic\ activity\ inhibition=100-\left(C/D\times 100\right) $$

where *C* is the absorbance value obtained for KM24 cultured on fish juice medium supplemented with sub-MICs of juniper EO and its major compounds and *D* is the absorbance value obtained for KM24 cultured on fish juice medium.

For the quantitative determination of the lipolytic activity of KM24, a modified method of Stuer et al. (1986) was used. Briefly, 10 ml of isopropanol containing 30 mg of *p*-nitrophenyl phosphate (*p*-NPP) (Sigma-Aldrich, Merck KGaA, Saint Louis, USA) was mixed with 90 ml of 0.05 M Sörensen phosphate buffer (Sigma-Aldrich, Merck KGaA, Saint Louis, USA), pH 8.0. The freshly prepared substrate solution (2.4 ml) was prewarmed at 37 °C and then mixed with 0.1 ml of the supernatant of the KM24 culture. After 15 min of incubation at 37 °C, the absorbance at 420 nm on a SPECORD® 205 UV-VIS spectrophotometer (Analytic Jena, AG, Jena, Germany) was read. The samples prepared with 0.1 ml of H_2_O served as controls. The percentage of lipolytic activity inhibition was calculated according to the following formula (3):

3 $$ \mathrm{Lipolytic}\ \mathrm{activity}\ \mathrm{inhibition}=100-\left(E/F\times 100\right) $$

where *E* is the absorbance value obtained for KM24 cultured on fish juice medium supplemented with sub-MICs of juniper EO and its major compounds and *F* is the absorbance value obtained for KM24 cultured on fish juice medium.

### RNA isolation, cDNA synthesis and RT-qPCR

The KM24 cultures were treated with RNAprotect® Bacteria Reagent (Qiagen, Valencia, CA, USA). A PureLink^TM^ RNA Mini Kit (Thermo Fisher Scientific, Waltham, MA, USA) and a PureLink^TM^ DNase Set (Invitrogen, Carlsbad, CA, USA) were used for total RNA isolation and purification. The quantity and quality of isolated RNA were estimated by the fluorescence-based Qubit^TM^ XR RNA and Qubit^TM^ IQ RNA Assay Kits (Thermo Fisher Scientific, Waltham, MA, USA) using a Qubit Fluorometer 4 (Thermo Fisher Scientific, Waltham, MA, USA). Total RNA (1.0 μg) and a High Capacity RNA-to-cDNA Kit (Life Technologies, Carlsbad, USA) were used to synthesize first strand +cDNA.

RT-qPCR analyses were carried out in a CFX96 Touch Real-Time PCR Detection System (BioRad, Hercules, CA, USA) using GoTaq® Master Mix (Promega, Walldorf, Germany). The primers used in this work are listed in Table 1. In the RT-qPCR experiments, the 16S rRNA gene was used as a reference gene. The following cycle conditions were applied: initial denaturation at 95 °C for 2 min and 45 cycles of denaturation at 95 °C for 15 s and annealing and extension at 58 °C for 1 min. The effect of sub-MIC concentrations of juniper EO, α-pinene, and sabinene on the changes in the expression of genes encoding QS autoinducer synthases, proteinases, and lipases in KM24 were calculated following the 2^−ΔΔCt^ method (Schmittgen and Livak 2008).

**Table 1:** The oligonucleotides applied in the RT-qPCR experiments

Primer	Sequence (5′→3′)	Amplified region
16S_F	GGAGACTGCCGGTGACAAAC	16S rRNA gene (universal primers)
16S_R	TGTAGCCCAGGCCGTAAGG	
LPTA_F	ACCTGGCTTACTTCGAAC	*lptA* gene
LPTA_R	ATGATGCGATTCTGCTGG	
LPTB_F	CACCGTCATTTGAACACC	*lptB* gene
LPTB_R	CCTTGTCCAGCCAGTATT	
PLSB_F	GTTCTTCTACCTCACGCC	*plsB* gene
PLSB_R	ATTTGTGCATTCTCTTCGG	
LIPA_F	CGAACTGCCGAAAAAACC	*lipA* gene
LIPA_R	CTGTGCAGCTTGTGTTTG	
LIPB_F	TGGTAAGCCGTATGGAGG	*lipB* gene
LIPB_R	CCGAGTGAGGCGATTTTC	
PRLC_F	AAAGCCAGGGCAAAAATC	*prlC* gene
PRLC_R	GTTACATACGGCGTTGAG	
CTPB_F	CAGAAAATCACCCTGACC	*ctpB* gene
CTPB_R	CTCTTCACGCTTTTGACC	

### Statistical analysis

Experiments were performed in triplicate. The results are presented as the means ± standard deviations. To characterize the difference between specific results, Tukey’s parametric *post hoc* test in Statistica software was used (Statsoft, Inc., 2012). Statistical significance was considered at *p* < 0.05.

## Results

### Protein and lipid contents in fish juice medium

To characterize the model growth environment for saprotrophic KM24, the contents of total protein and lipids were determined. The protein level of the examined medium was 1.8 mg g^−1^; the lipid content expressed on a wet weight basis was 0.635 mg g^−1^.

### RNA-Seq data processing and pfam analysis of differentially expressed CDSs/genes

In this work, the sequencing efficiency was 554.85 Mbp, and the cluster density was 97 K mm^−2^. The *Q* score value, which indicates the quality of the sequencing performed for 90.87% of the bases read, was 30. The percentage of passes filtered (PFs) for sequencing was 80.13%. Among 92.65% of the mapped readings, 91.38% were paired (Supplemental Table S1; Supplemental Fig. S1). The distribution of the distances between the ends of paired readings ranged from 50 to 150 bp. The number of CDS to which the readings were mapped was 1850; 1750 genes/CDSs were differentially expressed in KM24 cells incubated in fish juice medium. The reference sequences were prepared by an overlay with gene/CDS annotations using the Find Prokaryotic Genes tool. The conserved domains, gene names and functions of particular reading frames were assigned by the Annotate CDS with the Pfam Domains tool.

The transcribed/mapped genes/CDSs were screened with the criteria of the cellular/metabolic process category mainly associated with the QS system and the QS-mediated proteolytic and lipolytic activities of pseudomonads. The characteristics of the selected CDS of the pseudomonads transcriptome are presented in Table 2.

**Table 2:** The characteristics of the selected CDSs of *Pseudomonas fluorescens* transcriptome. Reference genome no. LS483372

Name	Region	Fragment per kilobase million value	Gene length	Description
*lptA*	1179239..1179814	47.00	576	Acyltransferase affect *quorum-sensing* signaling
*lptD*	5869389..5872214	10.77	2826	Acyltransferase affect *quorum-sensing* signaling
*plsB*	1553874..1556378	6.75	2505	Acyltransferase affect *quorum-sensing* signaling
*prlC*	52930..54981	23.08	2052	Metalloprotease
*ctpB*	424112..425428	23.12	1317	Protease
*lipA*	5678195..5679220	23.38	1026	Lipase
*lipB*	5679223..5679870	26.11	648	Lipase

Three of the mapped genes/CDSs were matched to the QS system: *lptA*, *lptD*, and *plsB*. These genes/CDSs encode acyltransferases that affect QS autoinducers. The FPKM value of *lptA*, *lptD*, and *plsB* was respectively: 47.0, 10.77, and 6.75, respectively. Two of the subsequently identified CDS, namely, *prlC* and *ctpB*, encode peptidases and *lipA* and *lipB* encode lipases in pseudomonads under in situ conditions. The FPKM values of *prlC* and *ctpB* were 23.08 23.12, respectively. The FPKM values of *lipA* and *lipB* were 23.38 and 26.11, respectively (Table 2).

### Chemical composition of juniper EO

The results of the GC/MS analysis described the chemical composition of juniper EO, and the relative percentages of 22 identified compounds are presented in Table 3. A TIC chromatogram of *J. phoenicea* L. EO on DB-5 column was presented on Supplemental Fig. S2. The major compounds comprising >8.0% of the total juniper EO volume were α-pinene (48.3%) and sabinene (8.4%). β-Pinene, α-terpinolene, and terpinen-4-ol were detected in minor amounts, ranging from 5.2 to 4.0% of juniper EO. The remaining compounds occurred in trace amounts (<4.0%). Individual juniper EO constituents categorized in the monoterpene group were dominant in the examined EO and accounted for 96.2% of the total volume; the presence of constituents of the sesquiterpene group did not exceed 3.8% of the total oil.

**Table 3:** Essential oil composition (%) of *Juniperus communis*

Compound	RI - Wax	RI - DB-5	Yield percentage (n-3)
α-Thujene	1021	938	0.8
α-Pinene	1035	939	48.3
Camphene	1080	953	3.5
β-Pinene	1118	980	5.2
Sabinene	1125	974	8.4
β-Myrcene	1158	990	3.4
α-Phellandrene	1170	1006	1.5
Limonene	1208	1030	1.6
β-Phellandrene	1245	1042	1.5
γ-Terpinene	1249	1072	1.5
*p*-Cymene	1274	1026	1.1
α-Erpinolene	1279	1083	4.0
Borneol	1506	1156	1.1
Linalol	1544	1100	0.9
Bornyl acetate	1584	1277	0.8
β-Caryophyllene	1594	1414	2.4
Terpinen-4-ol	1606	1180	4.5
α-Caryophyllene	1668	1449	2.8
α –Terpineol	1700	1192	1.5
Germacrene	1705	1487	1.0
Nerol	1792	1230	1.4
Geraniol	1852	1266	2.8

% Composition–the percentage composition calculated from the chromatogram obtained on the Supelcowax 10 column. Normalized peak area %. *RI–Wax*, retention index on Suplecowax 10 column; *RI- DB-5*, retention index on DB-5 column

### Minimum inhibitory concentration determination

To evaluate the antibacterial effect of selected dilutions of juniper EO, α-pinene, and sabinene against KM24, the buildup of biomass was monitored. In this study, concentrations below the sub-MIC of juniper EO (120 μl ml^−1^), α-pinene (40 μl ml^−1^), and sabinene (30 μl ml^−1^) were used for further experiments.

### Effect of sub-MICs of juniper EO, α-pinene, and sabinene on KM24 morphology

**Fig. 1:**
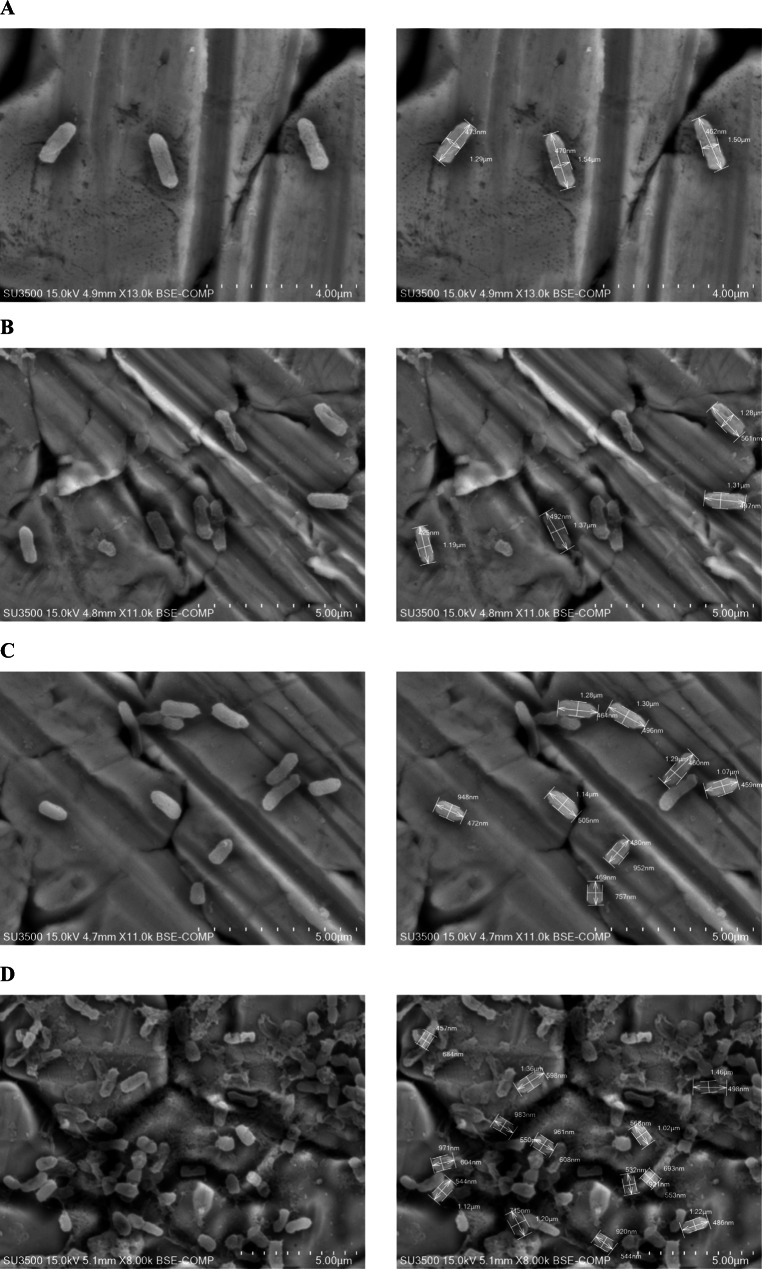
Microscopic images of *Pseudomonas fluorescens* KM24 cells grown on fish juice medium (**A**) supplemented with juniper essential oil (**B**), α-pinene (**C**), and sabinene (**D**) (magnification ranging from ×10000 to ×14000)

Microscopic comparison of KM24 reference cultures with KM24 cells treated with sub-MICs of juniper EO, α-pinene, and sabinene revealed changes in the morphology of cells, as shown in Fig. 1. Changes in cell dimensions, mainly in length from 1.19 to 1.37 μm for juniper EO, from 0.757 to 1.30 μm for α-pinene and from 0.684 to 1.46 μm for sabinene, were found. In the control, the length of cells ranged from 1.29 to 1.54 μm. The surface of untreated KM24 cells was smooth, the morphology was regular with an intact cell membrane, and individual bacteria were readily distinguishable (see Fig. 1).

### Effect of sub-MICs of juniper EO, α-pinene, and sabinene on quorum-sensing autoinducer synthesis

In this study, the pour plate method was first carried out to evaluate the antibacterial activity of sub-MICs of juniper EO, α-pinene, and sabinene toward the biosensor CV026 strain. No viability limitations of the biosensor strain at the examined concentrations of the tested agents were observed; therefore, the CV026 strain can be used as a biomarker for the presence of AHL (Supplemental Table S2).

**Fig. 2:**
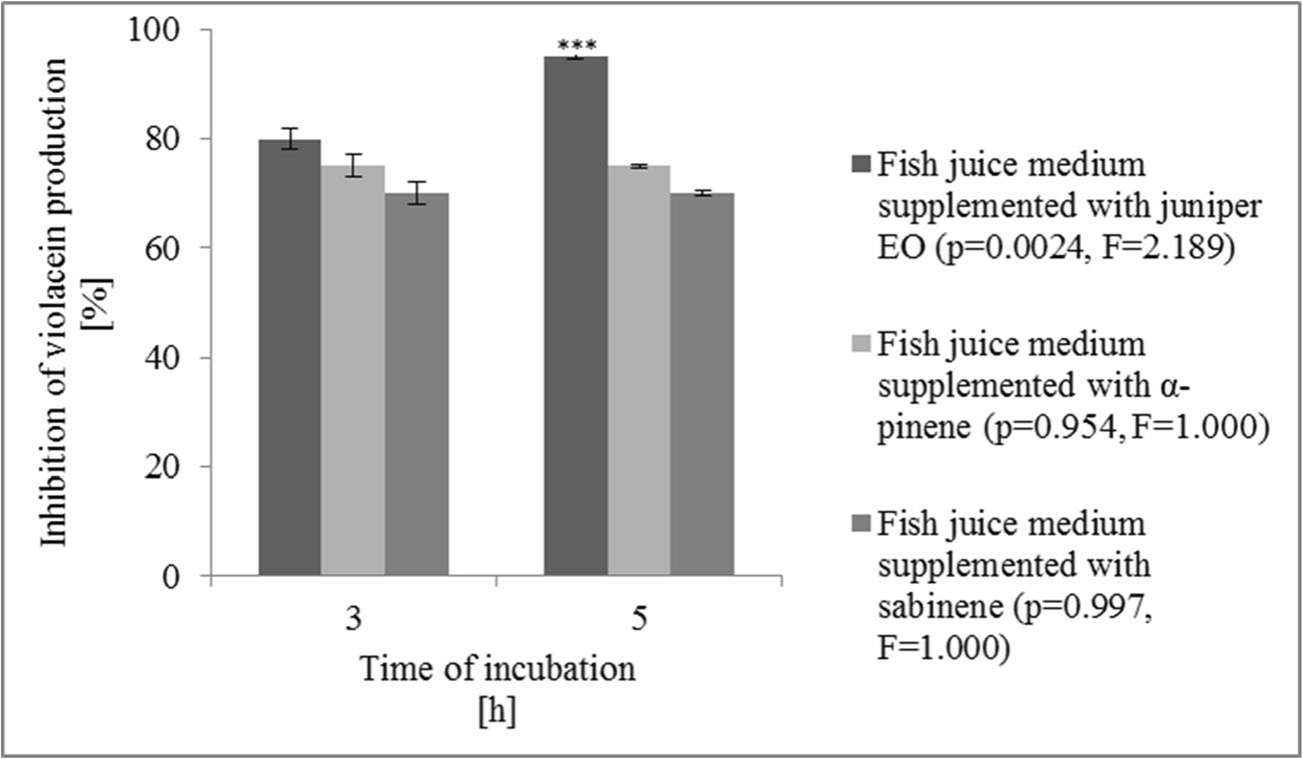
*Chromobacterium violaceum* CV026 cell response to AHL extract obtained from *Pseudomonas fluorescens* KM24 grown on fish juice medium supplemented with juniper essential oil, α-pinene, and sabinene. Also is shown is the significance of the experimental data determined by *t*-test (*p* < 0.005). Bars indicate standard deviation from three experiments

The Blosser and Gray (2000) assay was carried out to screen the anti-QS activity of juniper EO, α-pinene, and sabinene. In the assay, the pigmentation (purple-colored violacein production) of CV026 provided a naturally occurring phenotype in response to the presence of AHL with *N*-acyl side chains from C4 to C8 in length; limited AHL production by KM24 or low levels of AHL in fish juice medium inhibited violacein synthesis (Blosser and Gray 2000). Changes in AHL-related violacein production are shown in Fig. 2. The sub-MIC of juniper EO showed the highest anti-QS activity; in these experiments, a reduction of 80% at 3 days and 95% at 5 days of incubation in AHL-mediated violacein synthesis was noticed. Sub-MICs of α-pinene and sabinene caused reductions in violacein synthesis in the biosensor strain of approximately 75% and 70%, respectively, at all stages of cultivation examined (Fig. 2).

For further verification of the anti-QS activity of juniper EO, α-pinene, and sabinene at sub-MICs, the RP-UHPLC-ESI-MS system was used. The results of the experiments are presented in Table 4 and Supplemental Fig. S3. In the study, no evidence of QS autoinducers was found in samples collected from KM24 treated with all examined agents at all tested points. The profile of AHL of the reference KM24 culture at 5 days of cultivation consisted of 3-oxo-C14-HSL, 3-oxo-C6-HSL, C4-HSL, and *N*-[(RS)-3-hydroxybutyryl]-HSL. The concentrations of these molecules were: 0.570, 0.018, 3.744, and 0.554 μg ml^−1^, respectively. (see Table 4).

**Table 4:** RP-UHPLC-ESI-MS results of quality and quantity of quorum-sensing autoinducers produced by *Pseudomonas fluorescens* KM24 grown in fish juice medium enriched with juniper essential oil, α-pinene, and sabinene

Autoinducer (μg ml^−1^)	Growth conditions							
	Fish juice medium		Fish juice medium supplemented with juniper essential oil		Fish juice medium supplemented with α-pinene		Fish juice medium supplemented with sabinene	
	Time of incubation (days)							
	3	5	3	5	3	5	3	5
3-oxo-C14-HSL	< LOD^*^	0.570 ± 0.01	< LOD^*^	< LOD^*^	< LOD^*^	< LOD^*^	< LOD^*^	< LOD^*^
3-oxo-C6-HSL	0.014 ± 0.01	0.018 ± 0.0	< LOD^*^	< LOD^*^	< LOD^*^	< LOD^*^	< LOD^*^	< LOD^*^
C4-HSL	< LOD^*^	3.744 ± 0.15	< LOD^*^	< LOD^*^	<LOD^*^	< LOD^*^	< LOD^*^	< LOD^*^
*N*-[(RS)-3-hydroxybutyryl]-HSL	< LOD^*^	0.554 ± 0.01	< LOD^*^	< LOD^*^	< LOD^*^	< LOD^*^	< LOD^*^	< LOD^*^

< LOD^*^—under the limit of detection

Next, RT-qPCR experiments were carried out to gain insight into the molecular mechanism of the anti-QS activity of juniper EO, α-pinene, and sabinene at sub-MIC concentrations. In this work, mRNA expression of AHL synthase/acyltransferase genes in the KM24 strain was evaluated. KM24 cultivated in fish juice medium served as reference probes. The transcriptional changes in the *lptA*, *lptD*, and *plsB* genes were calculated as relative quantities normalized to the 16S rRNA gene.

*J. phoenicea* L. EO influenced the mRNA expression of all genes (i.e., *lptA*, *lptD*, and *plsB*) encoding acyltransferases (see Table 5); however, the highest reduction on the 3^rd^ and 5^th^ days of cultivation was observed for the *lptD* (−5.5 and −5.61, respectively) and *lptA* (−3.5 and −4.0, respectively) genes. In this study, the application of sub-MICs of α-pinene and sabinene to fish juice medium decreased the expression of QS autoinducer-related genes. The compounds downregulated all the genes evaluated at all the cultivation points, but the inhibition was only from −0.4 to −0.7 on average (Table 5).

**Table 5:** RT-qPCR confirmation of relative expression level of *quorum-sensing*-related genes of *Pseudomonas fluorescens* KM24 grown in fish juice medium enriched with juniper essential oil, α-pinene, and sabinene

Time of incubation (days)	Fish juice medium supplemented with juniper EO			Fish juice medium supplemented with α-pinene			Fish juice medium supplemented with sabinene		
	Log_2_ (relative quantity)								
	*lptA*	*lptD*	*plsB*	*lptA*	*lptD*	*plsB*	*lptA*	*lptD*	*plsB*
3	−3.5	−5.5	−1.5	−0.6	−0.5	−0.4	−0.4	−0.5	−0.6
5	−4.0	−5.61	−1.8	−0.7	−0.5	−0.5	−0.5	−0.5	−0.6

### Effect of sub-MICs of juniper EO, α-pinene, and sabinene on KM24 proteolytic activity

**Fig. 3:**
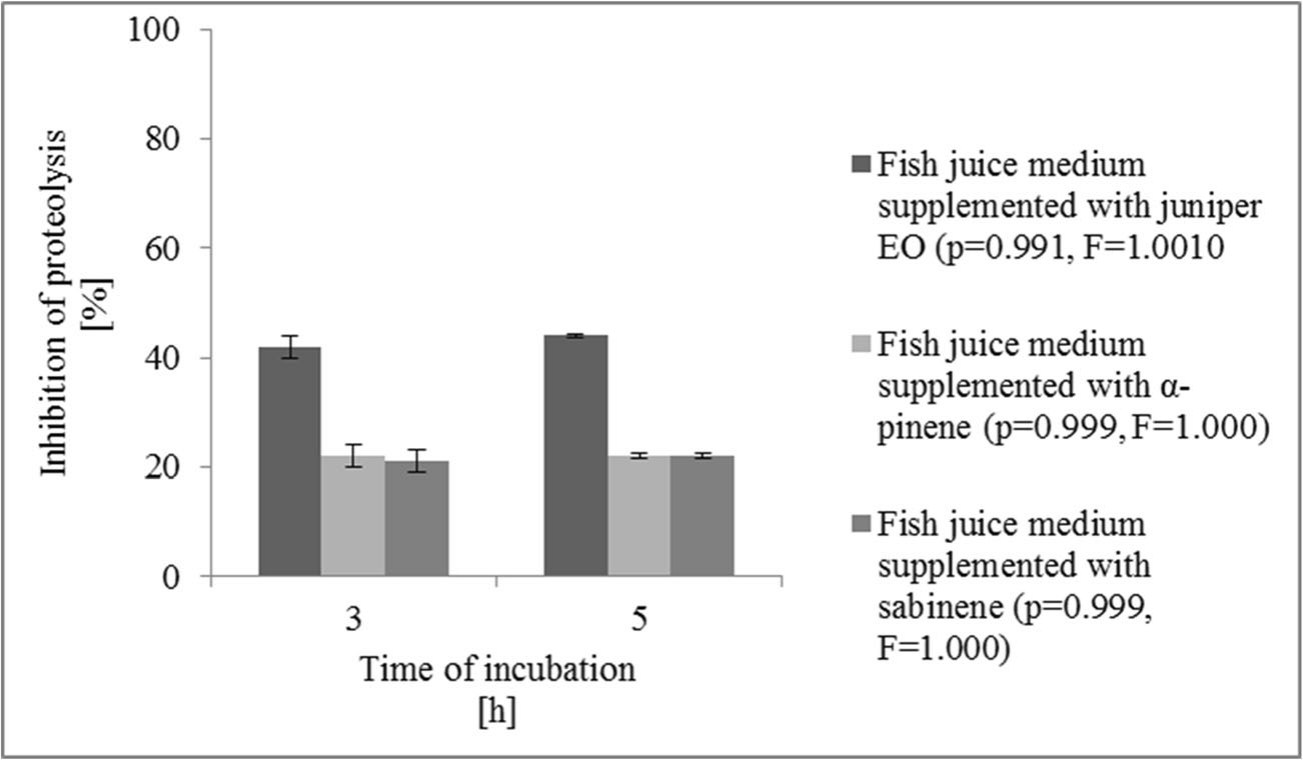
Inhibition of proteolytic activity of *Pseudomonas fluorescens* KM24 grown on fish juice medium supplemented with juniper essential oil, α-pinene, and sabinene. *t*-test showed no significant difference between experimental data (*p* < 0.005). Bars indicate standard deviation from three experiments

The changes in the proteolytic activity of KM24 caused by sub-MIC concentrations of juniper EO, α-pinene, and sabinene were investigated by spectrophotometric analysis with the TBNS reagent. The results of the percentage of inhibition of proteolysis after exposure of cells to sub-MICs of juniper EO and its major compounds are presented in Fig. 3. In this work, all of the agents used decreased the proteolytic activity of KM24 considerably (*p* < 0.05). The highest reduction was observed for pseudomonads treated with juniper EO (42%), while sabinene at sub-MIC concentrations had the lowest effect on the proteolytic activity of the bacteria examined (22%) (Fig. 3).

To extend the results obtained from the spectrophotometric measurements, the effects of juniper EO, α-pinene, and sabinene at sub-MICs on the expression of genes encoding proteases in KM24 were evaluated. As mentioned previously, the genes were indicated in the RNA-Seq experiment. In this work, the transcriptional levels of *prlC* and *ctpB* were normalized to the nondifferentially expressed reference 16S rRNA gene. As shown in Table 6, gene expression changed divergently; all the genes were downregulated by the applied treatments at all the examined time points. The highest reduction was observed for the *prlC* gene after treatment with sub-MICs of juniper EO. On the 3^rd^ and 5^th^ days of cultivation of the KM24 strain on fish juice medium supplemented with juniper oil, the mRNA level of *prlC* decreased to −3.5 and −4.2, respectively. α-Pinene and sabinene were also downregulated by all the proteases, but the degrees of change only ranged from −0.4 to −1.0.

**Table 6:** RT-qPCR confirmation of relative expression level of genes encoding proteases in *Pseudomonas fluorescens* KM24 grown in fish juice medium enriched with juniper essential oil, α-pinene, and sabinene

Time of incubation (days)	Fish juice medium supplemented with juniper EO		Fish juice medium supplemented with α-pinene		Fish juice medium supplemented with sabinene	
	Log_2_ (relative quantity)					
	*prlC*	*ctpB*	*prlC*	*ctpB*	*prlC*	*ctpB*
3	−3.5	−1.5	−0.4	−1.0	−0.5	−0.5
5	−4.2	−1.5	−0.5	−1.0	−0.5	−0.6

### Effects of sub-MICs of juniper EO, α-pinene, and sabinene on KM24 lipolytic activity

**Fig. 4:**
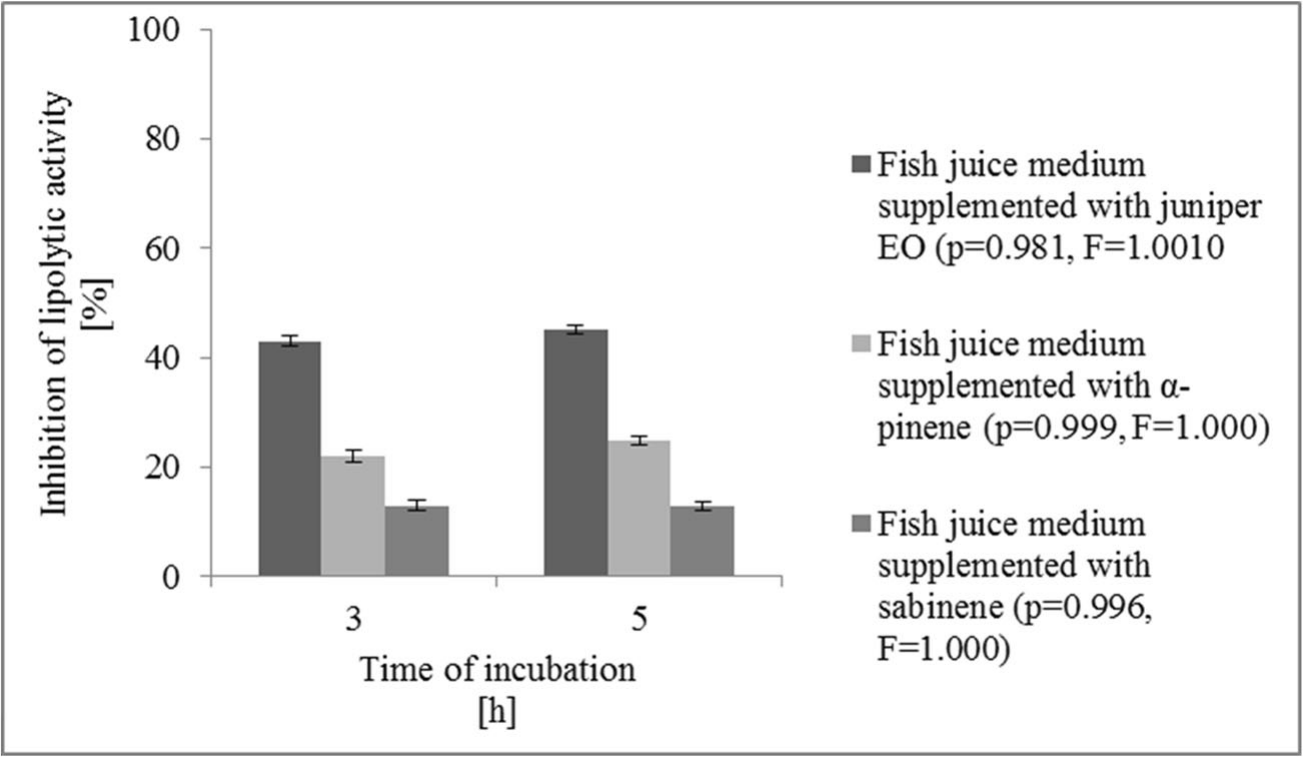
Inhibition of lipolytic activity of *Pseudomonas fluorescens* KM24 grown on fish juice medium supplemented with juniper essential oil, α-pinene, and sabinene. *t*-test showed no significant difference between experimental data (*p* < 0.005). Bars indicate standard deviation from three experiments

In this work, the spectrophotometric measurements employed by Stuer et al. (1986) and RT-qPCR experiments were performed to evaluate the effects of sub-MICs of juniper EO and its major compounds on the lipolytic activities of KM24. The addition of sub-MICs of juniper EO, α-pinene, and sabinene to fish juice medium inhibited lipase synthesis. However, the highest reduction in lipase production was recorded for pseudomonads treated with the oil (45%); α-pinene and sabinene at sub-MIC levels decreased bacterial lipase production to 25% and 13%, respectively (Fig. 4).

In this study, expression of the *lipA* and *lipB* genes confirmed the effect of the agents examined on the anti-lipolytic activities of KM24. Interestingly, regardless of the antimicrobial used, most gene expression was inhibited by between −0.4 and −0.6 on average (Table 7).

**Table 7:** RT-qPCR confirmation of relative expression level of genes encoding lipases in *Pseudomonas fluorescens* KM24 grown in fish juice medium enriched with juniper essential oil, α-pinene, and sabinene

Time of incubation (days)	Fish juice medium supplemented with juniper EO		Fish juice medium supplemented with α-pinene		Fish juice medium supplemented with sabinene	
	Log_2_ (relative quantity)					
	*lipA*	*lipB*	*lipA*	*lipB*	*lipA*	*lipB*
3	−0.6	−0.5	−0.3	−0.5	−0.5	−0.4
5	−0.6	−0.5	−0.5	−0.5	−0.6	−0.6

## Discussion

Live fish harbor cold-tolerant *Pseudomonas* spp., which can thus be considered the primary source of spoilage organisms on processed products (Ghaly et al.^ 2010^; Møretro et al. 2016). With an increasing number of reports of food spoilage incidents associated with cold-tolerant pseudomonads, solutions to prevent this phenomenon are urgently needed. *P. fluorescens* cultured at 4 °C exhibits higher spoilage potential than at the optimum temperature. The resistance traits of pseudomonads against conventional antimicrobials in food necessitate the search for alternative ways to manage bacterial metabolism and improve food quality and safety. Recently, the application of EOs has received much attention in the food industry due to their ecofriendliness, biodegradability, and mostly anti-QS properties (Tzortzakis and Economakis 2007). The stresses imposed by sub-MICs of EOs may interrupt autoinducer production and consequently downregulate QS-related enzymatic activity. However, the above hypothesis requires detailed verification under in situ conditions.

In this work, fish juice medium was used as a seafood-mimicking system to cultivate the wild-type KM24 strain. We determined the specific proportions of protein (1.8 mg g^−1^) and lipids (0.635 mg g^−1^) in the fish juice medium that did not differ from the average proportion of these components in marine salmon. The lipid content of marine salmon may vary between 0.58 and 7.83 mg g^−1^ (Zhang et al. 2020). The amount of protein in salmon muscle is usually somewhere between 1.5 and 2.0 mg g^−1^ (Tsuyuki et al. 1962). Fish juice medium provides pseudomonads with the right amount of nutrients and can thus simulate seafood.

In this study, RNA-Seq analysis revealed the physiological responses of the KM24 strain to the seafood ecosystem. In this work, transcribed genes/CDSs related to the QS process and enzymatic activity of the KM24 strain were unambiguously selected. The information obtained from RNA-Seq guaranteed the appropriate design of the RT-qPCR experiments. In this work, RNA-Seq analysis indicated key genes that are disrupted by food preservatives and may ultimately improve the quality and safety of fish-based products.

RNA-Seq analysis revealed three transcribed genes (i.e., *lptA*, *lptD*, and *plsB*) that are annotated as acyltransferase genes associated with the QS mechanism. Acyltransferase is an enzyme that transfers acyl groups to a target molecule. QS autoinducers contain an acyl moiety on their side chain, and the acyl groups of AHLs are a determinant of the specificity of QS autoinducers; appropriate transfer of acyl groups might be important in AHL synthesis, and acyltransferases can affect the profile of AHL production and possibly modulate QS signaling of bacteria (Yeom et al. 2013). *lptA*, *lptD*, and *plsB* encode a lysophatidic acid acyltransferase that catalyzes the second step of the biosynthetic pathway of phospholipids, which has been recognized to affect QS autoinducer production (Baysse et al. 2005). Baysse et al. (2005) revealed that a *lptA* mutation resulted in premature production of AHLs (mostly C4-HSL and C6-HSL) and repression of autoinducer synthesis at the stationary growth phase. The relationship between acyltransferase and QS was also reported in the study by Laue et al. (2000), where the acyltransferase of *P. fluorescens* F113 directly synthesizes AHLs. Additionally, the results of Yeom et al. (2013) suggest that acyltransferases play an important role in the QS regulation and expression of virulence factors.

In this work, systematic analysis of the RNA-Seq results also revealed two transcribed genes (namely, *prlC* and *ctpB*) involved in pseudomonads proteolysis in fish juice medium. *prlC* is involved in hydrolysis of oligopeptides that accumulate in large amounts in fish muscle (Venugopal 1990). The *ctpB* encodes an exoprotease that cleaves the fish protein substrate at the carboxyl terminus (Venugopal 1990). Secretion of both oligopeptidase A and exoprotease results in fish spoilage, which leads to the accumulation of volatile basic nitrogen compounds (Xie et al. 2018).

In this study, the global transcription profile of pseudomonads was also screened for genes/CDSs encoding lipases. *Pseudomonas* spp. lipases display thermoresistance and activity at alkaline pH. These traits are not common among lipases produced by other microorganisms, which highlights the importance of pseudomonad lipolytic activity in the seafood spoilage process (Martínez and Soberón-Chávez 2001). Similar to Dieckelmann et al. (1998), who investigated the diversity of lipases from psychrotrophic *P. fluorescens* C9, two transcribed genes, namely, *lipA* and *lipB*, which encode lipases, were detected in KM24 incubated in fish juice medium. The lipase encoded by *lipB* is solely responsible for the “lipolytic phenotype” of *P. fluorescens*, which leads to rancidity, a soapy off-flavor, and other quality defects of fish-based products (Beven et al. 2001). *lipA* is located at the end of a polycistronic operon in the *apr* gene cluster; its absence results in the loss of or relatively low lipolytic activities of *Pseudomonas* spp. (Woods et al. 2001).

Based on the work of Kerekes et al. (2013), EO from *Juniperus phoenicea* L. berries was used as an anti-QS agent toward KM24. In food industry systems, this oil reduces the formation of QS-related biofilms of foodborne *Bacillus cereus*, *Escherichia coli*, and *Campylobacter jejuni* (Kerekes et al. 2013; Šimunović et al. 2020). Moreover, fish-based products can be seasoned with juniper berries, through which seafood products obtain the taste desired by consumers (Montagne 1999).

In this study, the volatile constituents of juniper EO were determined by GC/MS analysis. Similar to the results of Höferl et al. (2014), high levels of representatives of the monoterpene group, i.e., α-pinene (48.3%) and sabinene (8.4%), were found in juniper oil. α-Pinene/sabinene-rich EO exhibits antimicrobial properties affecting microbial growth; with increasing concentrations of oil, the duration of the lag phase of bacterial growth may elongate (Tserennadmid et al. 2010; Majewska et al. 2017; Salehi et al. 2019). Tserennadmid et al. (2010) also revealed changes in the maximum specific growth rate parameter of *B. cereus* exposed to juniper EO. These changes may also influence AHL production (Jiang et al. 2014). Taking the aforementioned facts into account, in this work, sub-MICs of juniper EO, α-pinene, and sabinene were used as protective anti-QS agents against KM24, as concentrations above sub-MIC levels in foods could be sensorily unacceptable for consumers (Khan et al. 2009).

Determination of sub-MICs was performed by monitoring KM24 biomass buildup. In the study, concentrations of 120 μl ml^−1^, 40 μl ml^−1^, and 30 μl ml^−1^ of EO, α-pinene, and sabinene, respectively, were selected for further investigation. SEM experiments revealed alteration of the morphology of KM24 after exposure to sub-MICs of the examined agents. According to Di Pasqua et al. (2007), selective compounds of EOs may cause structural changes to the outer envelope of bacteria, causing the envelope to appear rougher. Terpenes from EOs often induce swelling of gram-negative bacteria (Nazzaro et al. 2013).

Next, we verified the anti-QS activity of juniper EO, α-pinene, and sabinene by the Blosser and Gray (2000) bioassay and an RP-UHPLC-ESI-MS system. All of the agents examined significantly inhibited QS autoinducer synthesis in the KM24 strain; at all the experimental time points, the agents affected CV026 pigment production and decreased the concentrations of all of the identified AHLs to levels lower than the limit of detection. In the reference culture at 5 days of incubation, the QS autoinducer profile of KM24 consisted of the following molecules: 3-oxo-C14-HSL, 3-oxo-C6-HSL, and *N-*[(RS)-3-hydroxybutyryl]-HSL, with C4-HSL as the major autoinducer whose content was specified at the level of 3.744 μg ml^−1^. The concentrations of 3-oxo-C14-HSL, 3-oxo-C6-HSL, and *N-*[(RS)-3-hydroxybutyryl]-HSL in the reference culture were 0.570, 0.018, and 0.554 μg ml^−1^, respectively. Based on the works of Guo et al. (2015) and Hawver et al. (2016), it can be concluded that suppression of the synthesis of AHLs with acyl chain lengths ranging from C4 to C6 in KM24 by juniper EO, α-pinene, and sabinene reduced violacein formation in the bioassay; however, inhibition of C4-HSL synthesis appears to be particularly significant. According to Swem et al. (2009) and Guo et al. (2015), C4-HSL can induce the maximum synthesis of violacein in CV026 in comparison to cognate AHLs. In contrast, AHLs with acyl chain lengths ranging from C10 to C14 are either weak antagonists or are completely inactive in the CV026 strain (Swem et al. 2009). In this work, the anti-QS activity of juniper EO, α-pinene, and sabinene at sub-MIC concentrations was also verified by RT-qPCR experiments. All examined agents influenced the mRNA levels of genes encoding acyltransferase in the KM24 culture. These results are in line with the observation of Alvarez et al. (2014), who also determined strong anti-QS properties of monoterpene-rich oregano EO. Selected concentrations of oregano EO inhibited violacein production and downregulated the *Cvil* gene encoding QS autoinducer synthase in *C. violaceum* (Alvarez et al. 2014). According to Deryabin et al. (2019), monoterpenes may reduce AHL synthesis both by affecting QS-related genes and by interacting with luxI-type proteins. The modeling data showed the interaction of plant-derived monoterpenes with acyltransferase with good stereochemical qualities (Deryabin et al. 2019; Sobieszczańska et al. 2020).

QS regulates proteolytic activity in *Pseudomonas* spp. (Sobieszczańska et al. 2020). This feature poses problems for the retention of quality seafood (Venugopal 1990). Several volatile odor-bearing compounds, such as volatile basic nitrogen compounds, volatile acids, H_2_S, and mercaptans, are produced in fish as a result of pseudomonad proteolytic activities (Xie et al. 2018). Our results showed that KM24 exhibited proteolytic activity at 4 °C. However, with low levels of AHLs, noticeably reduced protease synthesis and downregulation of the *prlC* and *ctpB* genes were observed. These observations agree with the work of Ahmed et al. (2019), who revealed that downregulation of the *lasI* gene encoding synthase AHLs in *Pseudomonas aeruginosa* resulted in a decrease in protease activity. The sub-MIC level of plant-derived QS inhibitors did not completely inhibit protease expression and production, but their administration significantly reduced this phenotype at both the transcriptional and extracellular levels in *P. aeruginosa* (Ahmed et al. 2019). These observations further support the view that EOs and their major compounds that target AHL synthesis can be potential food biocides for reducing bacterial spoilage activities.

Finally, the anti-lipolytic activities of juniper EO, α-pinene, and sabinene were evaluated. The lipolytic phenotype of *Pseudomonas* strains is also regulated by the QS system (Devescovi et al. 2007; Udine et al. 2013). Udine et al. (2013) revealed that supplementation of the culture medium with AHLs increased the transcription of *lipA* and *lipB* in *Burkholderia cenocepacia*. Additionally, the lipase encoded by *lipA* of *Burkholderia glumae* is regulated by AHL QS; the strain lost its capacity to synthesize lipase since it contained a nonfunctional AHL QS system (Devescovi et al. 2007). The results of this study confirmed that the lipolytic activities of KM24 lead to AHL-controlled phenotypes. The QS inhibitors examined reduced the lipolytic activities of KM24 in the fish-based model at all the tested points, while a sub-MIC of juniper EO was the most efficient. Our work directly proves that juniper EO can be used for the biopreservation of cold-stored fishery products.

In conclusion, fish from catch to consumption are prone to contamination with cold-tolerant *P. fluorescens*. The proteases and lipases of these bacteria pose problems for the retention of quality and safety during the storage of seafood. The application of conventional chilling and mechanical refrigeration is not adequate to protect fishery products against the action of bacterial proteases and lipases. The results of this work directly indicate that the use of α-pinene-/sabinene-rich juniper EO with anti-QS properties along with refrigeration could provide an additional advantage for controlling the spoilage activities of pseudomonads in fish.

## Supplementary Information

ESM 1 (PDF 880 kb)

## Data Availability

All data on which the conclusions were drawn are presented in this published article [and its electronic supplementary material].
