# Supplementary material for: In situ approaches show the limitation of the spoilage potential of Juniperus phoenicea L. essential oil against cold-tolerant Pseudomonas fluorescens KM24
Source: Appl Microbiol Biotechnol. 2021 May 14;105(10):4255–68. doi: 10.1007/s00253-021-11338-3 (PMC8140959; doi:10.1007/s00253-021-11338-3)
Supplement: Supplementary file 1 — (PDF 880 kb) [file 253_2021_11338_MOESM1_ESM.pdf]

Applied Microbiology and Biotechnology

In situ approaches show the limitation of the spoilage potential of *Juniperus phoenicea* L. essential oil against cold-tolerant *Pseudomonas fluorescens* KM24

Kamila Myszka[a,*], Natalia Tomaś[a], Łukasz Wolko[b], Artur Szwengiel[c], Anna Grygier[c], Katarzyna Nuc[b], Małgorzata Majcher[c]

[a] Department of Biotechnology and Food Microbiology, Poznan University of Life Sciences, Wojska Polskiego 48, Poznan, PL-60,627, Poland

[b] Department of Biochemistry and Biotechnology, Poznan University of Life Sciences, Dojazd 11, Poznan, PL-60-632, Poland

[c] Department of Food Technology of Plant Origin, Poznan University of Life Sciences, Wojska Polskiego 31, Poznan, PL-60-624, Poland

[*]corresponding author: e-mail address: kamila.myszka@up.poznan.pl (K. Myszka) ORCID ID 0000-0002-3574-3117

Table S1.

Mapped readings obtained from RNA-Seq for *Pseudomonas fluorescens*

	Number	Percentage of readings [%]	Average length [bp]
Mapped readings	619538	92.65	97.74
Non-mapped readings	49128	7.35	98.48
Mapped paired readings	610994	91.38	106.96

Table S2.

Impact of selected concentrations of juniper EO, α-pinene and sabinene on viability of *Chromobacterium violaceum* CV026. *t*-test showed no significant difference between experimental data (p=0.991; F=1.00) (p<0.005).

Cultivation variant	Log CFU ml^{-1}
TYB medium supplemented with sub-MIC of juniper EO	$6.0^{a} \pm 0.5$
TYB medium supplemented with sub-MIC of α-pinene	$5.8^{a} \pm 0.5$
TYB medium supplemented with sub-MIC of sabinene	$5.9^{a} \pm 0.2$
TYB medium	$6.1^{a} \pm 0.3$

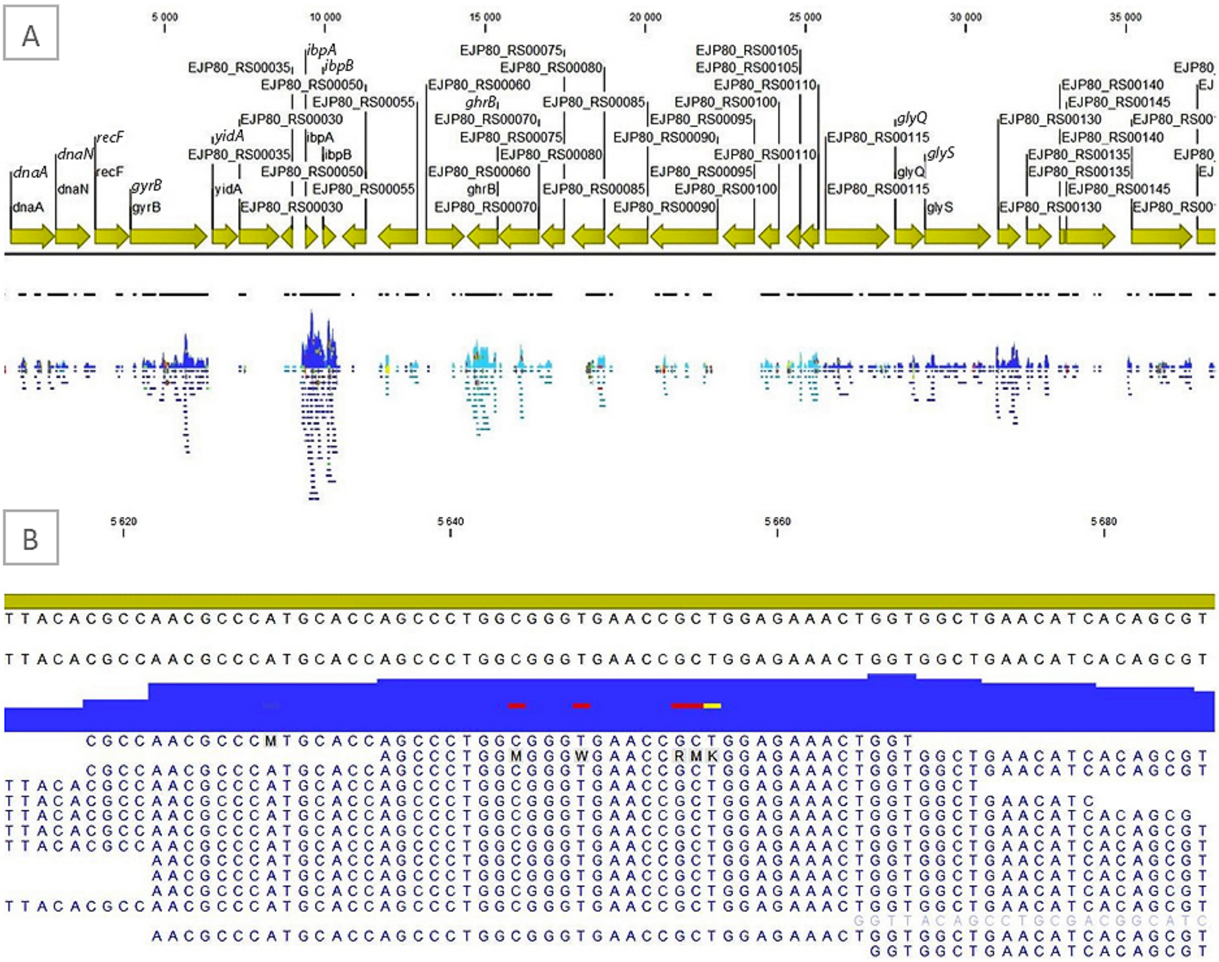

Fig. S1

An example of view of mapping of reads A-Differences in the level of gene transcription; B-The reference sequences and reads

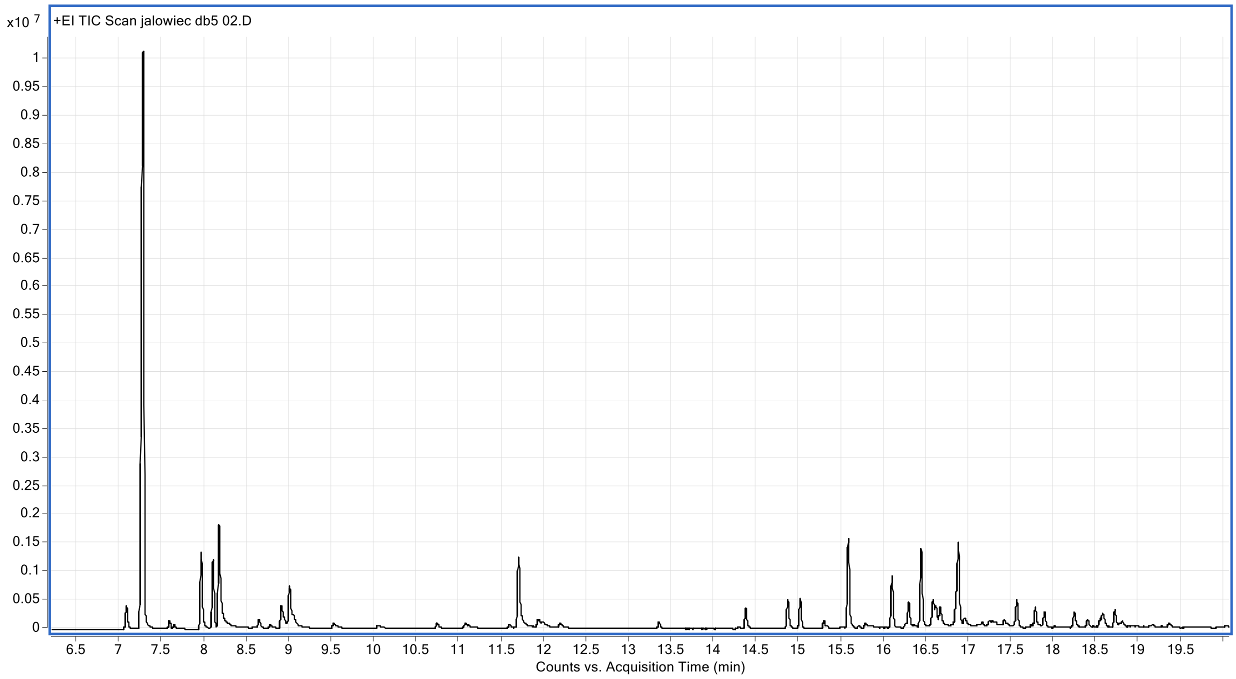

Fig. S2

TIC chromatogram of *Juniperus phoenicea* L. EO on DB-5 column

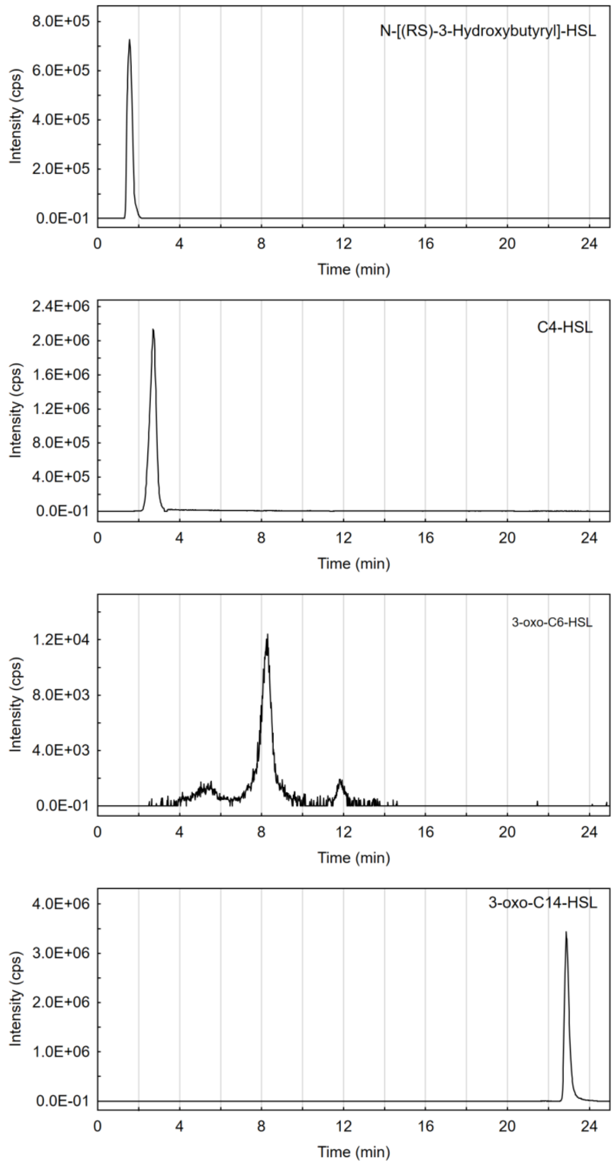

Fig. S3

Chromatograms of quorum sensing autoinducers produced by *Pseudomonas fluorescens* KM24 on 5th day of cultivation on fish juice medium
